# Performance of probe polymerization-conjunction-agarose gel electrophoresis in the rapid detection of KRAS gene mutation

**DOI:** 10.1590/1678-4685-GMB-2017-0197

**Published:** 2018-07-16

**Authors:** Na Xiao, Yi-Tong Tang, Zhi-Shan Li, Rui Cao, Rong Wang, Jiu-Ming Zou, Jiao Pei

**Affiliations:** 1 Medical College of Hubei University of Arts and Science, Xiangyang, China; 2 Department of Clinical Laboratory, Affiliated Hospital of Hubei University of Arts and Science, Xiangyang, China; 3 Department of Internal Medicine, Maternal and Child Health Care Hospital of Dongguan, Dongguan, China

**Keywords:** Mutation, circulating DNA, polymerization-conjunction reaction, agarose gel electrophoresis, K-ras

## Abstract

This study aimed to develop a simple and rapid method to detect KRAS gene mutations for conventional clinical applications under laboratory conditions. The genotype of mutation sites was determined based on the occurrence of target bands in the corresponding lanes of the reaction tubes through polymerization-conjunction of the probes, probe purification and amplification, and agarose gel electrophoresis. Circulating DNA samples were obtained from the plasma of 72 patients with lung cancer, which were identified based on six mutation sites (G12S, G12R, G12C, G12D, G12A, and G12V) of codon 12 of the *KRAS* gene. The detection results were compared with direct sequencing data. The proposed detection method is characterized by simple operation, high specificity, and high sensitivity (2%). This method can detect the mutations of three samples at G12S, G12R, and G12A. In the direct sequencing spectra of these samples, the genotype could not be determined due to the lack of evident sequencing peaks that correspond to the basic group of mutations. In conclusion, a simple and rapid method was established based on probe polymerization-conjunction-agarose gel electrophoresis for detecting KRAS gene mutations. This method can be applied to the conventional mutation detection of inhomogeneous samples.

## Introduction

The KRAS gene is a common oncogene in humans, and encodes the 21-kDa RAS protein. The RAS protein exhibits intrinsic guanosine triphosphate (GTP) enzymatic activity, and is involved in cell proliferation, differentiation, and apoptosis by switching the mutual transformation regulatory signal system between guanosine diphosphate (GDP) and GTP. The KRAS gene has a high mutation rate in patients with colorectal cancer and lung cancer, and confers the resistance to epidermal growth factor receptor (EGFR), tyrosine kinase inhibitors (TKIs), and EGFR monoclonal antibody agents (Chen *et al.*, 2013; Li *et al.*, 2014; Leiser *et al.*, 2015; Hsu *et al.*, 2016). Reports indicate that KRAS gene mutations in patients with non-small cell lung cancer are related to short survival time and poor prognosis (Li *et al.*, 2016; Tao *et al.*, 2016). The mutation rate of the KRAS gene significantly differs among various types of tumors and populations. For example, the mutation rate of the KRAS gene can reach as high as 15–30% in Caucasian patients with colorectal cancer, and only 4–10% in Asian patients with lung cancer (Mascaux *et al.*, 2005; Lu *et al.*, 2013; Kinugasa *et al.*, 2015; Omidifar *et al.*, 2015; Ohba *et al.*, 2016). Approximately 80% of KRAS gene mutations occur at codon 12, and the remaining mutations take place mainly in codons 13 and 61 (Mascaux *et al.*, 2005). Hence, the detection of KRAS gene mutations is important for the development of individualized treatments for patients with tumors, as well as to improve the effects of targeted clinical treatments and reduce treatment expenses.

KRAS gene mutations can be detected using various detection methods, including restriction fragment length polymorphism (RFLP) (Dobre *et al.*, 2013), DNA sequencing (Kinugasa *et al.*, 2015), capillary electrophoresis (Zhang *et al.*, 2013), high-resolution melting (HRM) curve analysis (Heideman *et al.*, 2009; Trujillo-Arribas *et al.*, 2016), mass spectroscopy (Choi *et al.*, 2014; Kriegsmann *et al.*, 2015), pyrosequencing (Altimari *et al.*, 2013; Vincenzi *et al.*, 2015; Mack *et al.*, 2016; Lee *et al.*, 2016), and ARMS real-time PCR (Hamfjord *et al.*, 2011; Zhang *et al.*, 2015). DNA sequencing is a traditional detection method that has low sensitivity. Pyrosequencing, HRM, and RT-PCR techniques exhibit high sensitivity, but also have high requirements in terms of experimental apparatus and conditions. These methods cannot be applied to conventional clinical detection under simple experimental conditions. In the present study, a simple, rapid, and sensitive method for detecting KRAS gene mutations was established based on probe polymerization-conjunction–agarose gel electrophoresis (PPC-AGE). This method was used to detect KRAS gene mutations at the six sites (G12S, G12R, G12C, G12D, G12A, and G12V) of codon 12.

## Materials and Methods

### Plasmid template and clinical samples

Plasmids that carry the wild and mutation alleles (G12S, G12R, G12D, and G12A) of codon 12 of the KRAS gene were constructed according to PCR-mediated site-specific mutagenesis technology. Plasma samples were collected from 72 patients with non-small cell lung cancer, who were admitted in the Affiliated Hospital of Hubei University of Arts and Science and Zaoyang Clinical College. Circulating DNA samples were extracted according to the instruction of the DNA extraction kit (QIAamp DNA Blood Mini Kit, Qiagen), and stored at -20°C for detection of KRAS gene mutations.

### Detection probe

**Figure 1:**
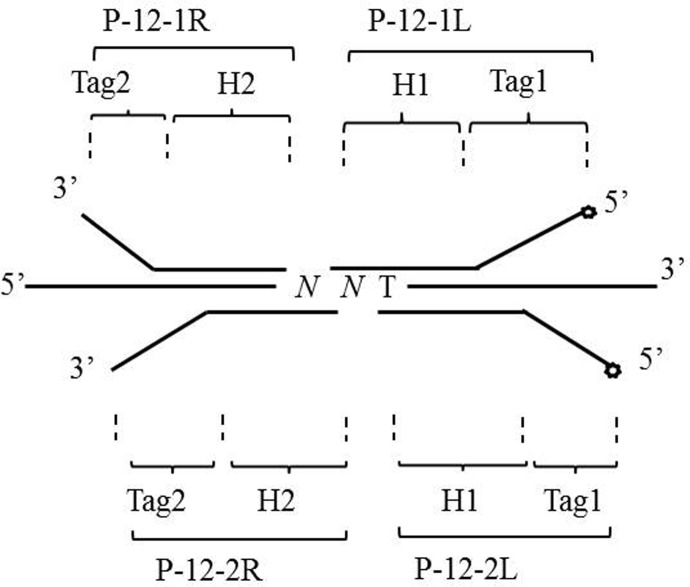
Design of the oligonucleotide probes. The open circle is the symbol of the biotin label. H1, H2, Tag1, and Tag2 represent the segments of the oligonucleotide probes. NNT represents the wild type and six mutation sites (G12S, G12R, G12C, G12D, G12A, and G12V) of codon 12 of the KRAS genes.

As shown in Figure 1, the mutations of codon 12 were detected using two pairs of oligonucleotide probes (P-12-1R/P-12-1L and P-12-2R/P-12-2L). P-12-1R and P-12-1L were used to detect mutations at G12S, G12R, and G12C, while P-12-2R and P-12-2L were used to test mutations at G12D, G12A, and G12V. Each probe pair comprises of five parts: homologous hybridization sequences (H1 and H2) with the template, general amplification primer sequences (Tag1 and Tag2), and the 5’-end biotin labeling of probes P-12-1L and P-12-2L. The oligonucleotide probe sequences were as follows:

P-12-1L: 5’biotin-GGGTTCGTGGTAGAGCGTCG GAGTACTCTTGCCTACGCCAC-3’,

P-12-1R: 5’p-AGCTCCAACTACCACAAGTGG CTGCTATCTCGGTGTCGTCTGG-3’;

P-12-2L: 5’biotin-GGGTTCGTGGTAGAGCGTC GGAGTCACTCTTGCCTACGCCA-3’;

P-12-2R: 5’p-CAGCTCCAACTACCACAAGGG CTGCTATCTCGGTGTCGTCTGG-3’.

Tag1 sequences and their sequences complementary with Tag2 (C Tag2) were as follows:

Tag1: 5’-GGGTTCGTGGTAGA GCGTCGGAGT-3’;

CTag2: 5’-CCAGACGACACCGAGATAGCAGC C-3’.

The oligonucleotide probes were synthesized by Shanghai Sangon Co., Ltd.

### KRAS gene amplification

Exon 2 of the KRAS gene was amplified by PCR. The forward and reverse primers used were as follows: 5’-TAAGCGTCGATGGAGGAGTT-3’ and 5’-CATCA TGGACCCTGACATAC-3’, respectively. The amplification system comprised of 5 μL of the circulating DNA sample, 2.5 μL (10 pmol/μL) of each amplification primer, 5 μL of deionized water, and 15 μL of 2× Taq PCR Mastermix. The amplification conditions were as follows: 32 cycles of 95°C for five minutes, 95°C for 45 seconds, 54°C for 45 seconds, 72°C for 90 seconds, and 72°C for four minutes. The amplified DNA sequences were extracted (SanPrep Column PCR Product Purification Kit, Shanghai Sangon Biotech) and stored at -20°C. The amplification products were subjected to the direct sequencing method.

### Polymerization and conjunction

P-12-1R and P-12-1L corresponded to a group of four reaction tubes (“G1,” “A1,” “C1,” and “T1”), and P-12-2R and P-12-2L to another group of four reaction tubes (“G2,” “A2,” “C2,” and “T2”). These eight reaction tubes were added with 28 μL of the reaction buffer (20 mM of Tris-HCl (pH 7.6), 25 mM of KAC, 10 mM of MgAC_2_, 10 mM of DTT, 1 mM of NAD, and 0.1% Trion-×100), 50 fmol of detection probes, and 5 fmol of template sequences. Next, the tubes were added with 1 μL of deoxyribonucleotide (25 μM) bases that are complementary with the labeling of different tubes (for example, dATP was added to tube T, and dCTP was added to tube G). These tubes were processed at 95°C for three minutes and at 49°C for five minutes. Subsequently, 1 μL of enzyme solution (1 unit Pfu of DNA polymerase (Shanghai Sangon, Co., Ltd., China) and 2 units of Taq DNA ligase (New England BioLabs, USA) were added. Then, the tube was again processed at 49°C for five minutes and at 98°C for 10 minutes. All reaction tubes were cooled in ice bath.

### Purification of the conjunction-dependent probe

Conjunction products in the different reaction tubes were purified through the magnetic particles of streptavidin (Xi’an GoldMag Nanobiotech Co. Ltd., China). These purified magnetic particles were washed by aqueous alkali (0.1 M of NaOH) to eliminate the non-specifically adsorbed nucleotide sequence. Finally, the magnetic particles were stored in Tris-HCl buffer solution (10 mM, pH 7.5).

### Amplification and detection

The conjunction-dependent probes that bound to the streptavidin magnetic beads were subjected to PCR amplification. A 20-μL reaction mix contained 5 μL of the magnetic particle template, 2.5 pmol of Tag1 and CTag2 primers, and 10 μL of 2 × *Taq* PCR MasterMix (Tiangen Co. Ltd., China). The reaction conditions were as follows: 95 °C for 30 s followed by 28 cycles of 95 °C for 25 s, 60 °C for 45 s, and 72 °C for 20 s, and 72 °C for 1 min. The PCR products were placed in different tubes and used for 3.5% agarose gel electrophoresis (AGE). The mutation detection results could be judged by the appearance of targeted detection fragments in the corresponding reaction tubes. For example, if the targeted band only appears in lanes corresponding to “G1” and “G2,” it is considered a wild template (the base sequence of codon 12 is GGT). If the targeted band appears in lanes corresponding to “G1,” “G2,” and “C1”, the template of codon 12 has the heterozygosis mutation of G12R (the base sequence of codon 12 is CGT/GGT). If the targeted band only appears in lanes corresponding to “C1”, the template of codon 12 has the homogenous mutation of G12R (the base sequence of codon 12 is CGT). The rest could be judged in the same manner.

## Results

### Detection flow

**Figure 2:**
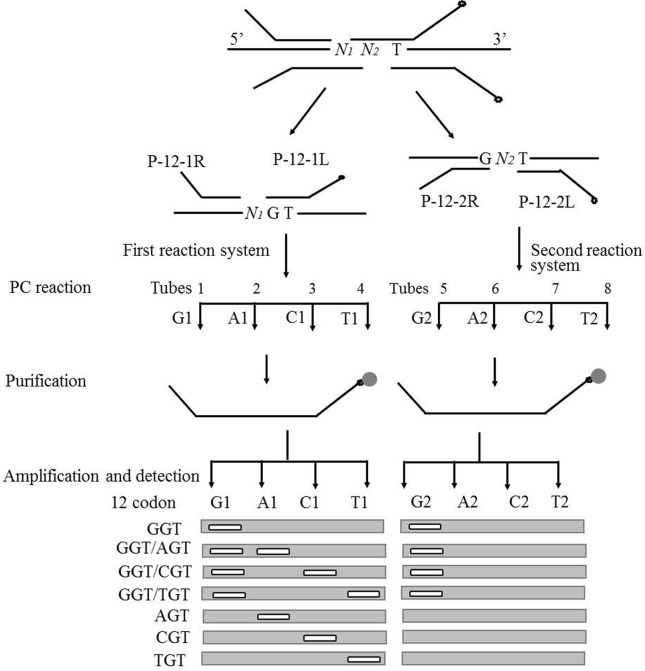
Detection assay for codon 12 of *K-ras*. Open circles represent the biotin label; closed circles are the symbol of streptavidin-coupled magnetic beads; *N1* and *N2* represent the bases of G, A, C, and T; G1, A1, C1, T1, and G2, A2, C2, T2 represent the four reactions corresponding to each pair of detection probes.

As shown in Figure 2, the entire detection process includes polymerization/conjunction reaction (PC reaction), purification, amplification, and detection. During the PC reaction, each pair of detection probes corresponds to the C, T, G, and A reaction tubes. After all reaction systems were heated and denaturated to the annealing temperature, gaps corresponding to the detected mutation base were formed among different probe pairs. Each deoxyribonucleotide was correspondingly added to different reaction tubes (for example, dATP was added to tube T and dCTP was added to tube G). When the added bases were complementary to the detection templates, the corresponding pair of detection probes was connected to the conjunction-dependent probes under the effect of DNA polymerase and ligase. However, these detection probes were not connected when the added base mismatched with the bases at the mutation sites of the template. After the PC reaction, the probes were purified using streptavidin magnetic particles. The conjunction-dependent probes were purified on the magnetic particle surface, and were used as a template. All purified products were amplified by PCR using Tag1 and CTag2. Then, the amplification products were subjected to 3.5% AGE. The mutation type was judged by the appearance of targeted bands in the corresponding lanes of the reaction tubes.

### Optimization of hybridization temperature

**Figure 3:**
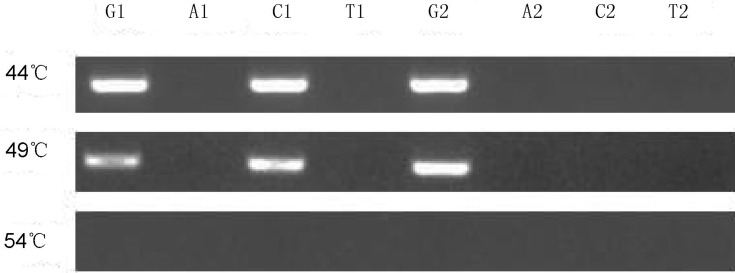
Optimization of hybridization temperature. G, A, C and T represent the four reactions corresponding to each pair of detection probes.

The annealing temperatures of P-12-1L (P-12-2L), P-12-1R (P-12-2R), and of the template ranged from 49 °C to 51 °C. The annealing temperature of the PC reaction was optimized using three temperature gradients: 44 °C, 49 °C, and 54 °C. The wild plasmid and mutation plasmid templates of G12R were used as detection objects. The AGE results are shown in Figure 3. At 44 °C and 49 °C, the amplification bands were clearly observed in lanes corresponding to tube C; the remaining lanes did not exhibit amplification bands. When the annealing temperature increased to 54 °C, all lanes had no amplification bands. This finding shows that the annealing ability of probes on the detection template is negatively correlated with annealing temperature. The detection probes could be annealed to the template from 44 °C to 49 °C, in order to obtain the conjunction reaction. However, these probes could not be annealed to the template at temperatures higher than 54 °C due to the absence of targeted amplification bands. Hence, 49 °C was selected as the optimal temperature to achieve an effective annealing of probes on the template and a specificity of the PC reaction.

### Specificity of detection

**Figure 4:**
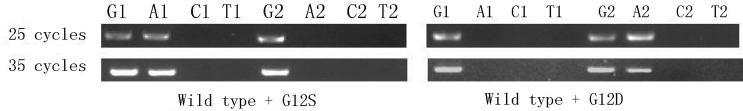
Specificity detection with the PCR cycle numbers. G, A, C and T represent the four reactions corresponding to each pair of detection probes.

The specificity of the proposed detection method was tested by determining the cycle number in the amplification reaction. Equal proportions of wild plasmid template and mutation plasmid template (G12S or G12D) were mixed, and the cycle number of the amplification reaction was set to 25 and 35. Approximately 5 μL of the amplification products were collected from each reaction tube and used for 3.5% AGE (Figure 4). Under two circulation conditions, the wild-type and mutation plasmids of G12S, P-12-1L, and P-12-1R corresponded to G1, A1, C1, and T1 reactions. Obvious bands were detected in lanes corresponding to G1 and A1. In addition, P-12-2L and P-12-2R corresponded to the G2, A2, C2, and T2 reactions, and an obvious band was detected in the lane corresponding to G2. Concerning wild and G12D mutations, an obvious band was detected in lanes corresponding to G1, G2, and A2. Meanwhile, nonspecific bands in other lanes were absent for 35 cycles. Hence, the proposed detection method exhibits high specificity and amplification volume.

### Sensitivity test

**Figure 5:**
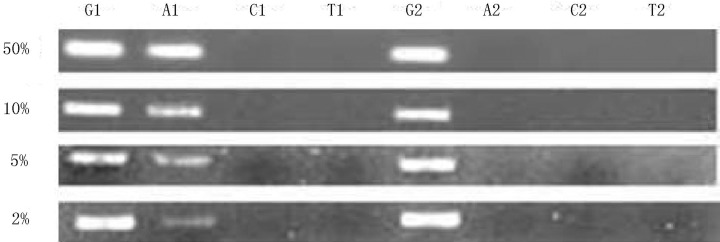
Sensitivity detection with agarose gel electrophoresis. The proportions of mutated alleles in the total plasmid DNA were 50, 10, 5 and 2%. G, A, C and T represent the four reactions corresponding to each pair of detection probes.

Different proportions of wild plasmid and mutation plasmid templates (G12S) were mixed. The proportions of the mutation plasmid template in the total detection template were set to 50, 10, 5, and 2% (Figure 5). Under all proportions of the mutation plasmid templates, amplification bands were found in lanes corresponding to C and T. With the reduction of the mutation allele (G12S), amplification bands in lanes corresponding to T darkened. However, amplification bands were still detected in lanes corresponding to T when the proportion of the mutation allele was lower than 2%. This finding shows that the proposed method exhibits high sensitivity, and that it can be applied to detect mutation alleles in inhomogeneous samples.

### Sample detection

**Figure 6:**
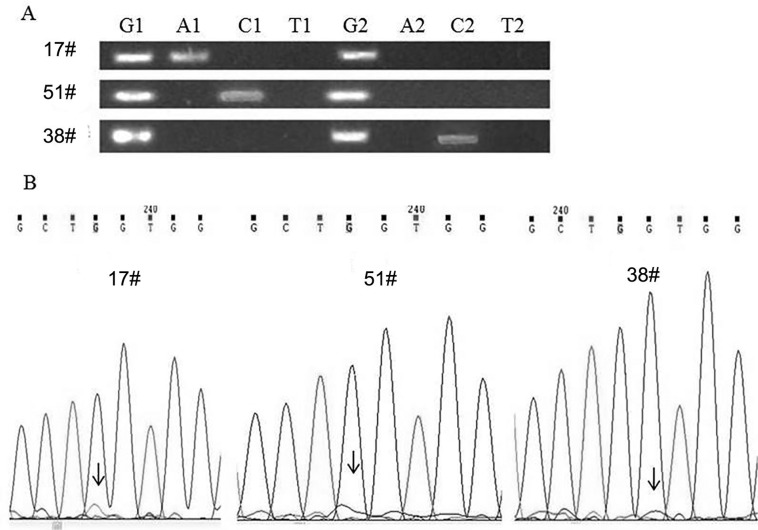
Mutation detection of the clinical samples. (A) The results of agarose gel electrophoresis for codon 12 of the K-ras gene. (B) Results of the direct sequencing. G, A, C and T represent the four reactions corresponding to each pair of detection probes.

The mutations of codon 12 of the KRAS gene in 72 patients were detected using the proposed method. The detection results were compared with the direct sequencing data (Figure 6A,B). Mutations were detected in three samples: the G12S (GGT/AGT) mutation of the #17 sample, the G12R (GGT/CGT) mutation of the #51 sample, and the G12A (GGT/GCT) mutation of the #38 sample. However, these direct sequencing results did not exhibit obvious sequencing peaks corresponding to mutation bases at codon 12 of the KRAS gene. Impure peaks corresponding to mutation bases were found in #17, #51, and #38 samples, and almost overlapped with the background peak. Therefore, determining the genotype according to direct sequencing results alone is difficult. These results confirm that the proposed method is superior to direct sequencing in terms of its sensitivity and applicability for detecting inhomogeneous samples.

## Discussion

The present study established a simple and fast method for KRAS gene mutation detection based on the method for detecting EGFR gene mutations (Tang *et al.*, 2014). The proposed detection method can detect six kinds of mutation behaviors of codon 12 through eight reactions, as well as the mutation behaviors of codons 13 and 16 using the same principle. This method is simpler and does not need expensive experimental instruments or reagents, such as fluorescent probes and high-quality detection samples, when compared to methods like fluorescent quantitative PCR, mass spectrometry, or DNA direct sequencing. The probe length is less than 50 bp, and it can detect mutation alleles using ordinary PCR instruments and AGE. Compared with the RFLP method, ours has no limitations on the enzyme cutting site and is more applicable for mutation detection under simple and conventional experimental conditions.

The high specificity of the proposed method can be maintained by: (1) using one pair of probes and four similar reaction systems, when detecting one single-base mutation site. Each reaction system was added with one dNTP to ensure that the PC reaction could be accomplished only upon complementation between the added dNTP and the base at the mutation site of the detection template. In the four reaction systems, the PCR amplification results of two systems could be used to determine the genotype of the mutation site, and the remaining reaction systems could be used as a control group to avoid non-specific results. (2) The PC reaction can only be accomplished under the dual specificity of DNA polymerase and DNA ligase. (3) The Tm values of H1 and H2 of the detection probe differed by 11 °C compared with that of Tag1 and Tag2 in the amplification primers, thereby reducing the mutual influence between the connection reaction and the follow-up amplification reaction.

This method exhibits high detection sensitivity and can detect as low as 2% mutation alleles in the mixed plasmid template where wild/mutation sites are located. Therefore, the proposed method can be used to detect mutations in inhomogeneous samples, design Tag1 and Tag2 on the probe sequence, and implement the secondary amplification of products after PC reaction in order to increase their numbers, resulting in a fast detection under the AGE level. Although DNA sequencing is the gold standard of mutation detection, it exhibits low sensitivity (only 20–50%; Garcia *et al.*, 2000; Dufort *et al.*, 2009), which is inadequate for detecting gene mutations in tumor tissues, circulating DNA samples, and other inhomogeneous samples. In the present study, KRAS mutations in the samples could not be judged based on the sequencing spectra alone, because the sequencing peaks that corresponded to the mutation sites in the direct sequencing spectra could not be separated from the background signal. Pyrosequencing, RT-PCR, and mass spectrum detection methods exhibit sensitivity as high as 1% (Jarry *et al.*, 2004; Dufort *et al.*, 2009), but require an expensive detection platform, equipment and reagents. Hence, these techniques cannot be applied under conventional laboratory conditions.

In conclusion, a method for detecting KRAS gene mutations was established based on PPC-AGE. This method can be easily operated, and is applicable for inhomogeneous samples under simple experimental conditions (e.g., ordinary nucleic acid electrophoresis and PCR equipment). Furthermore, this method does not need expensive experimental apparatuses and reagents. However, it cannot detect unknown mutation sites, and the detection sensitivity of AGE is limited. Therefore, this method cannot be applied when the total proportion of mutation alleles in inhomogeneous samples is lower than 2%. The proposed method has limited detection capabilities.

## References

[B1] Altimari A, de Biase D, De Maglio G, Gruppioni E, Capizzi E, Degiovanni A, D’Errico A, Pession A, Pizzolitto S, Fiorentino M (2013). 454 next generation-sequencing outperforms allele-specific PCR, Sanger sequencing, and pyrosequencing for routine KRAS mutation analysis of formalin-fixed, paraffin-embedded samples. Onco Targets Ther.

[B2] Chen J, Bi H, Hou J, Zhang X, Zhang C, Yue L, Wen X, Liu D, Shi H, Yuan J (2013). Atorvastatin overcomes gefitinib resistance in KRAS mutant human non-small cell lung carcinoma cells. Cell Death Dis.

[B3] Choi HS, Min KT, Cha YS, Hong SP (2014). Multiplex detection of KRAS mutations by a matrix-assisted laser desorption/ionization-time of flight mass spectrometry assay. Clin Biochem.

[B4] Dobre M, Comanescu M, Arsene D, Iosif C, Bussolati G (2013). K-ras gene mutation status in colorectal cancer: Comparative analysis of pyrosequencing and PCR-RFLP. Rom J Morphol Embryol.

[B5] Dufort S, Richard MJ, de Fraipont F (2009). Pyrosequencing method to detect KRAS mutation in formalin-fixed and paraffin-embedded tumor tissues. Anal Biochem.

[B6] Garcia CA, Ahmadian A, Gharizadeh B, Lundeberg J, Ronaghi M, Nyrén P (2000). Mutation detection by pyrosequencing: sequencing of exons 5-8 of the p53 tumor suppressor gene. Gene.

[B7] Hamfjord J, Stangeland AM, Skrede ML, Tveit KM, Ikdahl T, Kure EH (2011). Wobble-enhanced ARMS method for detection of KRAS and BRAF mutations. Diagn Mol Pathol.

[B8] Heideman DA, Thunnissen FB, Doeleman M, Kramer D, Verheul HM, Smit EF, Postmus PE, Meijer CJ, Meijer GA, Snijders PJ (2009). A panel of high resolution melting (HRM) technology-based assays with direct sequencing possibility for effective mutation screening of EGFR and K-ras genes. Cell Oncol.

[B9] Hsu HC, Thiam TK, Lu YJ, Yeh CY, Tsai WS, You JF, Hung HY, Tsai CN, Hsu A, Chen HC (2016). Mutations of KRAS/NRAS/BRAF predict Cetuximab resistance in metastatic colorectal cancer patients. Oncotarget.

[B10] Jarry A, Masson D, Cassagnau E, Parois S, Laboisse C, Denis MG (2004). Real-time allele-specific amplification for sensitive detection of the BRAF mutation V600E. Mol Cell Probes.

[B11] Kinugasa H, Nouso K, Miyahara K, Morimoto Y, Dohi C, Tsutsumi K, Kato H, Matsubara T, Okada H, Yamamoto K (2015). Detection of K-ras gene mutation by liquid biopsy in patients with pancreatic cancer. Cancer.

[B12] Kriegsmann M, Arens N, Endris V, Weichert W, Kriegsmann J (2015). Detection of KRAS, NRAS and BRAF by mass spectrometry-a sensitive, reliable, fast and cost-effective technique. Diagn Pathol.

[B13] Lee SE, Lee SY, Park HK, Oh SY, Kim HJ, Lee KY, Kim WS (2016). Detection of EGFR and KRAS mutation by pyrosequencing analysis in cytologic samples of non-small cell lung cancer. J Korean Med Sci.

[B14] Leiser D, Medová M, Mikami K, Nisa L, Stroka D, Blaukat A, Bladt F, Aebersold DM, Zimmer Y (2015). KRAS and HRAS mutations confer resistance to MET targeting in preclinical models of MET-expressing tumor cells. Mol Oncol.

[B15] Li T, Zheng Y, Sun H, Zhuang R, Liu J, Liu T, Cai W (2016). K-Ras mutation detection in liquid biopsy and tumor tissue as prognostic biomarker in patients with pancreatic cancer: a systematic review with meta-analysis. Med Oncol.

[B16] Li W, Shi Q, Wang W, Liu J, Ren J, Li Q, Hou F (2014). KRAS status and resistance to epidermal growth factor receptor tyrosine-kinase inhibitor treatment in patients with metastatic colorectal cancer: a meta-analysis. Colorectal Dis.

[B17] Lu SS, Xu X, Guo HQ, Cao J, Pan QJ, Wang MR (2013). Detection of EGFR and K-ras mutations in non-small cell lung cancer using cytological specimens. Zhonghua Zhong Liu Za Zhi.

[B18] Mack E, Stabla K, Riera-Knorrenschild J, Moll R, Neubauer A, Brendel C (2016). A rational two-step approach to KRAS mutation testing in colorectal cancer using high resolution melting analysis and pyrosequencing. BMC Cancer.

[B19] Mascaux C, Iannino N, Martin B, Paesmans M, Berghmans T, Dusart M, Haller A, Lothaire P, Meert AP, Noel S (2005). The role of RAS oncogene in survival of patients with lung cancer: A systematic review of the literature with meta-analysis. Br J Cancer.

[B20] Ohba T, Toyokawa G, Osoegawa A, Hirai F, Yamaguchi M, Taguchi K, Seto T, Takenoyama M, Ichinose Y, Sugio K (2016). Mutations of the EGFR, K-ras, EML4-ALK, and BRAF genes in resected pathological stage I lung adenocarcinoma. Surg Today.

[B21] Omidifar N, Geramizadeh B, Mirzai M (2015). K-ras mutation in colorectal cancer, a report from Southern Iran. Iran J Med Sci.

[B22] Tang YT, Xiao N, Li ZS, Zou JM, Cao R, Zhao XH, Shao JH (2014). Detection of mutations by fill-in ligation reaction with enzyme-linked immunosorbent assay for rapid medical diagnosis. Biosci Biotech Bioch.

[B23] Tao LY, Zhang LF, Xiu DR, Yuan CH, Ma ZL, Jiang B (2016). Prognostic significance of K-ras mutations in pancreatic cancer: a meta-analysis. World J Surg Oncol.

[B24] Trujillo-Arribas E, Macher HC, Jiménez-Arriscado P, de la Portilla F, Molinero P, Guerrero JM, Rubio A (2016). Screening of KRAS mutation in pre- and post-surgery serum of patients suffering from colon cancer by COLD-PCR HRM. Adv Exp Med Biol.

[B25] Vincenzi B, Cremolini C, Sartore-Bianchi A, Russo A, Mannavola F, Perrone G, Pantano F, Loupakis F, Rossini D, Ongaro E (2015). Prognostic significance of K-Ras mutation rate in metastatic colorectal cancer patients. Oncotarget.

[B26] Zhang H, Song J, Ren H, Xu Z, Wang X, Shan L, Fang J (2013). Detection of low-abundance KRAS mutations in colorectal cancer using microfluidic capillary electrophoresis-based restriction fragment length polymorphism method with optimized assay conditions. PLoS One.

[B27] Zhang H, Zhang X, Wang J, Xian J, Chen X, Zhang W (2015). Comparison of high-resolution melting analysis, Sanger sequencing and ARMS for KRAS mutation detection in metastatic colorectal cancer. Clin Lab.

